# The surprising subtleties of changing fear memory: a challenge for translational science

**DOI:** 10.1098/rstb.2017.0033

**Published:** 2018-01-29

**Authors:** Merel Kindt

**Affiliations:** Department of Clinical Psychology, University of Amsterdam, Nieuwe Achtergracht 129B, 1018WS, Amsterdam, The Netherlands

**Keywords:** emotional memory disorders, fear memory, reconsolidation, extinction, translational science

## Abstract

Current pharmacological and psychological treatments for disorders of emotional memory only dampen the affective response while leaving the original fear memory intact. Under adverse circumstances, these original memories regain prominence, causing relapses in many patients. The (re)discovery in neuroscience that after reactivation consolidated fear memories may return to a transient labile state, requiring a process of restabilization in order to persist, offers a window of opportunity for modifying fear memories with amnestic agents. This process, known as memory reconsolidation, opens avenues for developing a revolutionary treatment for emotional memory disorders. The reconsolidation intervention challenges the dominant pharmacological and psychological models of treatment: it is only effective when the amnestic drug is given in conjunction with memory reactivation during a specific time window, and a modification of cognitive processes is a boundary condition for changing fear. Notwithstanding the dramatic effects of targeting memory reconsolidation in the laboratory (i.e. proof of principle), the greatest hurdle to overcome is that the success of the manipulation depends on subtle differences in the reactivation procedure. These experimental parameters cannot be easily controlled in clinical practice. In harnessing the clinical potential of memory reconsolidation, a heuristic for *bi-directionally translating* behavioural neuroscience and clinical science is proposed.

This article is part of a discussion meeting issue ‘Of mice and mental health: facilitating dialogue between basic and clinical neuroscientists’.

## 1. Introduction

While many of our precious memories fall into oblivion, fear memories are typically strong and resistant to decay. The general failure of forgetting emotional memories may in some respects be evolutionarily advantageous, but the robustness of fear memories can be a double-edged sword in patients suffering from emotional memory disorders: the disorders that have sparked the quest for a means of *therapeutic forgetting*.

Since the time of the ancient Greeks, people have imagined memories to be a stable form of information, as if they are indelible portraits of our past. The metaphors for this persistence have changed over time: from impressions in a wax tablet by Plato, to computer analogies that are still popular today. The most widely accepted view in science was that memories were only initially labile and sensitive to disruption (short-term memory), after which they became fixed or ‘consolidated’ into the physical architecture of the brain (long-term memory) [1]. At the turn of this century, a major breakthrough in neuroscience was achieved with the (re)discovery that fear memory is not inevitably permanent,^1^ but can change when retrieved [2,3]. Nader and colleagues demonstrated in rats that, upon a reminder cue, consolidated memories may return to a labile state, requiring de novo protein synthesis for restabilization in order to persist [2]. This process is now referred to as the ‘memory reconsolidation hypothesis’ and offers a window of opportunity for targeting fear memories with amnestic agents. Over the past decade, evidence for persistently reducing fear response by pharmacologically interfering with the process of memory reconsolidation has progressed from animals to humans [4]. In a series of laboratory experiments, we convincingly demonstrated that disrupting the process of memory reconsolidation with a drug neutralized the affective expression of a fear memory without changing the actual recollection of the threatening event [4–13]. A technology that instantaneously dampens the emotional impact of unduly intense fear memories would signify a true paradigm shift in the practice of psychotherapy. Instead of multiple sessions of cognitive behavioural treatment or daily drug intake with a gradual and often temporary decline of symptoms [14], it involves one single instance of treatment that leads to a sudden—albeit delayed—decline in fear [5,15]. The reconsolidation intervention is furthermore in stark contrast with a fundamental tenet of cognitive behavioural therapy (CBT), a well-established treatment for disorders of emotional memory. The CBT model postulates that a change in cognitive processes (i.e. threat beliefs) is a condition sine qua non for obtaining a treatment effect [14,16], while a cognitive change is not required for the reconsolidation intervention. In contrast, when a cognitive change takes place during the reconsolidation intervention, this actually poses a boundary condition for the treatment [8,17,18]. The reconsolidation intervention also represents a shift in the use of pharmacological agents to alleviate symptoms. It involves just one single administration of a very common drug (i.e. 40 mg propranolol HCl) administered during a specific time window. In other words, the procedure is more like ‘neurosurgery’ than psychotherapy. Although these findings suggest the possibility of a paradigm shift in clinical practice, a reconsolidation intervention for horrific or otherwise undesirable memories has not left the hypothetical arena. Until now, an abrupt reduction in fear response using a single amnestic drug administered upon memory reactivation has only been reliably demonstrated for very specific fears, which are typically induced in the laboratory, and more recently in a subclinical sample of people with spider fear [4–13,15]. Irrespective of these dramatic effects (i.e. proof of principle), the results of the reconsolidation intervention for more severe emotional memory disorders such as chronic post-traumatic stress disorder (PTSD) are both promising and disappointing [19–23]. It is worth noting, however, that the swift translation from basic to clinical science could easily bypass several important steps [23,24]. Laboratory experiments in animals and humans illustrate that the effect of the reconsolidation intervention depends on subtle differences in the reactivation procedure in interaction with the learning history [8,25], while these experimental parameters cannot be easily controlled in clinical practice. In harnessing the clinical potential of memory reconsolidation as an alternative treatment for emotional memory disorders, a *bi-directional translational approach* is warranted. Not only are fundamental insights on the principles of learning and memory from the animal and human literature indispensable, clinical observations need to be considered as well. These insights and observations provide operational tools for testing novel hypotheses on different levels of analysis: from behavioural neuroscience to clinical science, and vice versa.

## 2. Emotional memory and psychopathology

Although there is a growing interest in discovering transdiagnostic processes to understand the aetiology and treatment of mental disorders (e.g. Research Domain Criteria initiative of the National Institute of Mental Health), clinical science is still oriented toward the Diagnostic and Statistical Manual of Mental Disorders (DSM) in which diagnoses are based on symptom counts nominated by clinical consensus. The claim made here is that emotional memory is at the root of a broad range of mental disorders, from anxiety disorders and PTSD to addiction and eating disorders [26,27]. In daily life, emotional memory is generally used to indicate the recollection of specific emotional events, while in neuroscience it refers to a much broader taxonomy of subjective, behavioural, physiological and neural mnemonic outputs, with the common denominator that they reflect emotional learning in the recent or distant past.

The focus of this paper is on fear memory, which is obviously most relevant for the treatment of anxiety disorders and PTSD [26,28]. These disorders often do not result from direct fear learning experiences such as traumatic events; they may also result from indirect or vicarious learning experiences [29]. Yet, irrespective of the learning history, fear and anxiety disorders are represented in the brain as associative memory networks [30]. The leading model for studying associative fear learning and memory is Pavlovian fear conditioning, which has proven to be an excellent paradigm across a wide range of organisms in the laboratory. It involves the repeated pairing of an initially neutral or ambiguous cue (e.g. a tone or picture as conditioned stimulus, CS) with an inherently aversive stimulus (e.g. an electric shock as unconditioned stimulus, US). As a result, the CS and US representation will become connected in the brain. Evidence for memory consolidation is based on later retention tests where re-exposure to the CS elicits a conditioned fear response (e.g. freezing in rats, potentiated startle reflex in humans). Insofar as associative fear memory is regarded as the core of emotional memory disorders [26,28], it does not only entail contingency learning where the originally neutral or ambiguous stimulus (CS) becomes a valid predictor for a negative experience (US). The feared stimulus also becomes imbued with the affective and motivational properties of the reinforcers (US) they predict, which influences behaviour in a number of powerful ways [31].

**Figure 1.**
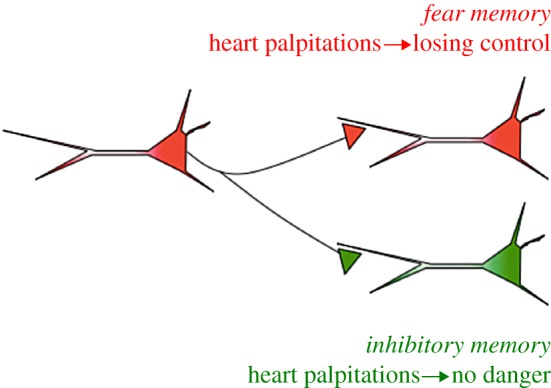
The *fear memory* involves physical sensations like heart palpitations that are associated with the feeling of losing control or going crazy. The heart palpitations trigger panic attacks and anticipatory fear of having panic attacks in the future. During exposure treatment patients with panic disorder are repeatedly exposed to heart palpitations without experiencing the feared outcome. As a consequence an *inhibitory memory* is formed and the fear of heart palpitations and panic attacks gradually subsides.

In addition to its utility in understanding the formation and consolidation of fear memory, the Pavlovian fear-conditioning paradigm has also proven to be a suitable translational model for developing and advancing treatment for emotional memory disorders [26,28,31–33]. Fear extinction is one of the most extensively studied procedures for reducing learned fear. It involves a decrement in conditioned fear responding that occurs with repeated presentation of a conditioned fear stimulus without the aversive consequence (i.e. unreinforced). As a result, the conditioned stimulus gains new predictive properties. Although extinction-like exposure treatments (i.e. *in vivo* or imaginary confrontations with the feared object without the expected adverse consequences) are among the most effective strategies to assuage excessive fears, there are many patients who experience a relapse even after initially successful treatment [14,34]. Explanations for the return of fear after CBT-like interventions can be found in the behavioural neuroscience literature on associative fear learning and memory. Fear-conditioning research in animals and humans has reliably shown that subsequent to extinction training, the conditioned fear response can easily return through memory retrieval techniques: re-exposure to unsignalled USs (i.e. reinstatement), a context change (i.e. renewal), testing several weeks later (i.e. spontaneous recovery) or accelerated re-acquisition [33,35–37]. Together, these phenomena constitute strong evidence that extinction does not erase the original fear memory, but it rather reflects the formation of a new association (CS/no US) that inhibits expression of the fear memory. The extinction memory competes for behavioural control at retrieval, while the fear response driven by the original fear memory is only temporarily suppressed. Even after successful fear extinction, the fear memory remains intact and may resurface, resulting in a return of fear [35–37] (figure 1). The implication for clinical practice is that, irrespective of the potential of extinction training as a means of therapeutic forgetting, the fear symptoms may easily return when patients are confronted with an unexpected aversive situation, when leaving the therapeutic exposure context, or simply with the passage of time.

## 3. Paradigm shift in changing fear memories

The discovery that fear memory in animals is not necessarily permanent but can change when reactivated may therefore be a promising alternative for the treatment of emotional memory disorders [24,26]. Nader *et al.* [2] showed in their landmark study that infusion of a protein synthesis inhibitor (i.e. anisomycin) shortly after memory reactivation produced post-reactivation amnesia. Noticeably, short-term fear memory (4 h) was still intact after memory reactivation and drug administration, but the long-term expression of fear memory (24 h) was strongly reduced. Consistent with a time-limited role for protein synthesis in consolidation, delaying the drug infusion until 6 h after memory reactivation produced no amnesia. This process known as memory reconsolidation refers to two phases by which previously consolidated memories (i) transfer to a transient destabilized state upon reactivation and (ii) require a time-dependent restabilization to persist. Gene transcription, RNA and protein synthesis are necessary for this restabilization and offer a window of opportunity for either pharmacological or behavioural interventions to neutralize the fear memory [38]. Despite the compelling evidence for memory reconsolidation in the laboratory, an obvious limitation in human memory research is that the neurobiological processes of memory destabilization and restabilization can be neither directly observed nor locally targeted. Hence, the critical conditions for targeting the process of memory reconsolidation in patients with emotional memory disorders are still largely unknown.

**Figure 2.**
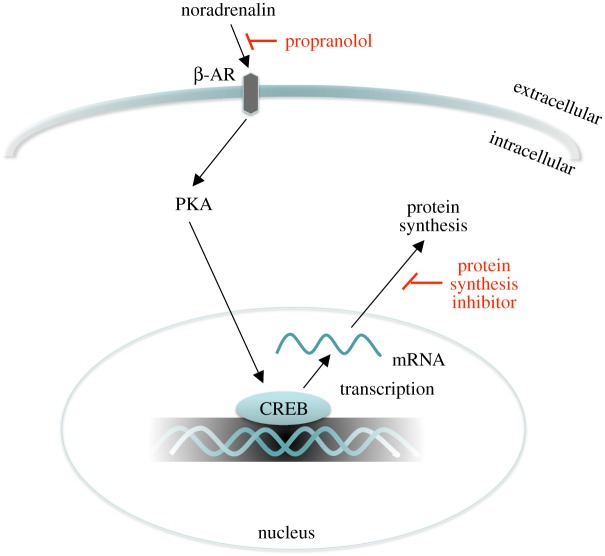
Propranolol, a lipophilic β-AR antagonist that crosses the blood–brain barrier, targets β-ARs in the amygdala. β-ARs play an essential role in protein synthesis via the downstream β-AR/PKA/CREB signalling pathway, one of the molecular cascades that regulates the gene transcription and subsequent protein synthesis required for the consolidation and reconsolidation of memory [42–44]. Nadolol, a hydrophilic β-AR antagonist (i.e. does not cross the blood–brain barrier), did not produce post-reactivation amnesia in humans [5].

The most convincing studies showing post-reactivation amnesia in animals used protein synthesis inhibitors (i.e. anisomycin) infused locally into the amygdala, a key brain area of fear conditioning [2,38]. Given the toxicity of protein synthesis inhibitors, there is no translational feasibility for using these drugs in humans. However, at the time our first reconsolidation experiment was designed, there were also a few successful demonstrations of post-reactivation amnesia in animals with an alternative pharmacological agent: the β-adrenergic receptor (β-AR) antagonist propranolol, which was infused either systemically or locally into the amygdala [39,40]. Even after systemic infusion, the effects of propranolol on reconsolidation are achieved by targeting the amygdala [40], and disruption of fear memory reconsolidation is correlated with a reduction of synaptic potentiation in the lateral amygdala selective to the reactivated fear memory [41] (figure 2).

Propranolol could potentially be interesting for studying post-reactivation amnesia in humans as it can be marked as a nontoxic drug when taken in a low dose and only once. Our research demonstrated that one pill of propranolol (40 mg) administered *prior to* or *after* memory reactivation effectively neutralized the fear-conditioned startle response and prevented the return of fear 1 day/month later [4–13]. The repeated observation that memory retrieval techniques did not lead to the re-emergence of fear memory expression, as is generally observed after extinction training, supports the superiority of disrupting reconsolidation as a means of therapeutic forgetting. Importantly, the fear-erasing effects of propranolol only occurred for the memory traces that were reactivated [4,5,9–11] and only when administered within a specific time window upon the reminder cue [5]. Hence, the fear reduction cannot be explained by a general fear-dampening effect of the drug, indicating that the reconsolidation intervention is noticeably different from traditional pharmacological treatments.

In the past 15 years there has been a wealth of demonstrations conveying memory reconsolidation across species (including roundworms, crabs, chicks, honeybees, rodents and humans), learning tasks and amnestic agents. Although these findings suggest that memory reconsolidation is a fundamental process [38], there are also experimental conditions under which reconsolidation does not seem to occur. For instance, animal research indicates that the success of memory reconsolidation is dependent on both the age and strength of memories, with older and stronger memories becoming increasingly resistant to disruption [45]. These conditions are subsequently viewed as boundary conditions of reconsolidation, but evidence for boundary conditions is based on negative findings with a *single reactivation procedure* [46]. There is, however, no single, universally effective reactivation procedure that always induces reconsolidation. For instance, remote or strongly trained memories may be rendered labile by longer reminder sessions [47,48] or by exposure to novel stimuli at reactivation [49]. Strong memories can also undergo reconsolidation if they are reactivated at remote time-points [50,51]. In light of these findings, it has also been postulated that true boundary conditions for reconsolidation do actually not exist [38,52]. This is in line with the alleged evolutionarily conserved role of reconsolidation as a way to keep memories up-to-date, should conditions require such adaptation [53]. As such, even repeated trials of the same training procedure could induce reconsolidation as long as the reactivation procedure involves a discrepancy or a match/mismatch experience between what has already been learned and what can be learned (i.e. *Prediction Error*, PE) [54–56]. The concept of PE is founded on the work of Rescorla & Wagner [57], who postulated that surprise will dictate when learning occurs. Likewise, we observed in humans that mere retrieval is not sufficient to induce memory destabilization and subsequent restabilization. Instead, memory reconsolidation is triggered *only if* the reactivation contains surprising, but relevant, information [6].

The idea of a crucial role of PE in inducing reconsolidation was launched by Pedreira and colleagues [54], who reported a necessary role for PE in memory reconsolidation. Since then, PE has been used in several experimental settings, across different species, to trigger reconsolidation [18]. However, the occurrence of PE in *animals* could only be inferred from the modified memory expression in a later retention test. In our *human* studies, the amnestic agent (i.e. propranolol) presented before or after memory reactivation specifically affected the emotional expression of fear memory (i.e. fear-potentiated startle response), while the cognitive level (i.e. US-expectancy ratings) remained unaffected [4–13]. By capitalizing on this striking dissociation between the cognitive and emotional expression of fear memory, we developed a measure of PE that is independent from the observation of fear memory reconsolidation [7,18]. In particular, we demonstrated in a fear-conditioning study that reconsolidation was induced by reminder conditions that led to a subsequent change in ratings of US expectancy upon CS presentation (i.e. match/mismatch). Reconsolidation occurred whenever the delivery (positive PE) or omission (negative PE) of an aversive stimulus (US) was not fully predicted by the CS that was presented during reactivation. In contrast, reminder conditions that left reported US expectancies unaffected did not induce fear memory reconsolidation [7]. Despite these new insights on PE as a necessary condition for triggering memory reconsolidation, there are several issues that pose a major challenge for translating the process of memory reconsolidation to clinical practice.

## 4. A challenge for translational science

If PE is a necessary condition for triggering memory reconsolidation, this does not imply that PE is also a sufficient condition. PE can also give rise to new learning (e.g. extinction learning) [57] and it has been shown that extinction training puts a constraint on reconsolidation [17,58,59]. During extinction training, repeated or prolonged unreinforced exposure generates multiple PEs, which eventually reduce both the threat expectancy and fear response. Modern exposure treatment is designed to enhance new learning by disconfirming expectancies regarding the temporal latency, frequency and intensity of negative outcomes [14]. Sometimes the expected outcome is an ‘inability to tolerate discomfort’ or ‘going crazy’, in which case the exposure is designed to test out the ability to accomplish the exposure task despite high levels of fear. For the reconsolidation intervention, a violation of threat expectancy is also necessary, but with the difference that it should not lead to new learning [18]. If the aim of exposure is to trigger the process of memory reconsolidation, then it should be much shorter and occur only once as opposed to traditional CBT. Of relevance is the observation in humans that even before extinction of the fear response can be observed physiologically, boundary conditions for memory reconsolidation may have already been reached [8]. While changes in cognition are assumed to precede behavioural modifications in CBT, a slight decrease of threat expectancy during the reconsolidation intervention demarcates this boundary [8,18]. Parallel observations in rodents suggest that reconsolidation and extinction not only are mutually exclusive, but also seem to be separated by a transitional phase (i.e. limbo state), insensitive to amnestic agents, presumably because neither of the two opposite processes of memory reconsolidation or extinction is engaged [25]. Consequently, a specific reactivation may be either too similar (i.e. retrieval) to or too dissimilar (i.e. limbo or extinction) from the original memory to trigger reconsolidation. Minor environmental changes define whether reactivation induces retrieval, memory reconsolidation, a limbo state or the initiation of a new inhibitory memory trace. The implication for clinical practice is that the window of opportunity for targeting emotional memory with amnestic agents is small. It is preceded and followed by phases that leave the original memory unaffected.

A further challenge in translating reconsolidation to clinical practice is that there is no single universal reactivation for inducing memory reconsolidation. PE is not simply governed by the reminder session, but by the interaction between the original learning and the reminder session [7,8]. In a fear-conditioning study, the interaction between the original learning experience and the reminder can be relatively easily manipulated, given that the threat contingency during acquisition and memory reactivation is under experimental control. In clinical practice, however, the learning history of people suffering from emotional memory disorders generally involves many instances of direct or indirect learning experiences, of which patients are often not aware. If we are to design a reconsolidation intervention in clinical practice, we need to establish whether a certain reminder session actually triggers memory reconsolidation.

Germane to this issue is the lack of real-time assessment of PE as a potential marker to induce memory reconsolidation. In the fear-conditioning studies, the PE could only be inferred from a change in reported threat predictions measured from the end of fear acquisition to the retention test the following day. This confirms previous findings on aversive learning in the crab *Chasmagnathus* showing that reconsolidation does not start at CS onset, but is triggered by CS offset, when the occurrence or non-occurrence of the anticipated threat becomes evident (i.e. match/mismatch or PE) [54]. An obvious shortcoming of the current assessment of PE in a fear-conditioning setting is that the threat predictions during exposure to the (un)reinforced CS correspond with a new learning experience. As a consequence, the effect of the CS offset during memory reactivation could only be revealed 1 day later in a retention test. In our future research we aim to develop a real-time marker for memory destabilization independent from the individual learning history. This would enable us to optimally design the memory reactivation for individual patients during the intervention itself (e.g. elongate the exposure).

## 5. Memory reconsolidation in clinical practice

In a recent study we have successfully translated the laboratory findings on conditioned fear response to a subclinical trial in individuals with spider phobia. We showed that a single intervention of 40 mg of the β-AR blocker propranolol HCl (double-blind/placebo-controlled) upon a very brief memory reactivation transformed avoidance behaviour into approach behaviour in a virtually binary fashion; an effect that persisted at least 1 year post-treatment [15]. Importantly, the fear-reducing effect was not restricted to the phobic stimulus of the intervention, but generalized to other reminder cues. We also demonstrated that the change in fear behaviour could not be explained by a general fear-dampening effect of propranolol or by an exposure effect, because the abrupt change in fear behaviour was only observed when the active drug (propranolol/placebo control) was given in conjunction with memory reactivation.

To exemplify the heuristic of a *bi-directional translational approach* in harnessing the clinical potential of a reconsolidation intervention, the unfolding of the reconsolidation intervention for spider fear is presented here in extraordinary detail. Drawing from a range of insights on the necessary and boundary conditions for inducing memory reconsolidation, we inferred that the exposure to the feared cue (i.e. spider) ought to be very brief, but the exact duration of the exposure was still indefinable. Even though the discovery of PE as a necessary condition for inducing memory reconsolidation is informative, the index for measuring PE in the laboratory is inappropriate for a direct translation into clinical practice. To trigger memory reconsolidation, the participants were instructed to approach a tarantula in a terrarium and to touch it, after which they received 40 mg of propranolol or a placebo. As well as being at the far end of the threat continuum, tarantulas can be easily controlled in an experimental setting. They usually sit still in the terrarium and only start moving after they are sprinkled with water. In the first series of pilot cases, the exposure durations varied noticeably between the participants. Although a few participants with subclinical spider fear were actually able to very briefly touch the tarantula, many of them were not and became extremely distressed. Most importantly, the treatment effects were absent or small and not specific for the active drug. In the next series of pilot cases, the participants were only instructed to approach the spider, while the instruction to touch the spider was left out. Although the exposure duration was under experimental control this time, a reactivation by only approaching a tarantula in a terrarium was not sufficiently threatening for the subclinical participants. In one further series of pilot cases, the participants were instructed that they would be asked to touch a tarantula, but after a brief 2 min exposure to the tarantula—and without actually touching the spider—the experimenter closed the terrarium. The participants were then seated next door, where they received an oral dose of 40 mg of propranolol HCl. Given the compelling observations in several pilot cases, this procedure was eventually tested in the randomized controlled trial (double-blind/placebo-controlled) in a new sample of individuals with subclinical spider fear [15].

Notwithstanding the success of this reconsolidation intervention in changing avoidance behaviour into approach behaviour, the distinctive features of the reactivation session that actually triggered the process of memory reconsolidation remain elusive. Given that all participants profited from the intervention, the lack of variation in the outcome prohibited inferring an index of PE on the basis of their cognitive and emotional expression of fear. Obviously, not the general success of the intervention, but the lack of a valid index for assessing PE in a clinical context has to be addressed. It is worth noting here that the false information regarding the instruction to touch a spider was not meant to trigger PE, but to maximally reactivate the fear memory, while the unexpectedness of the procedure could be easily conceived as a manipulation of PE. Previously, we postulated that a PE should be relevant to the fear memory [6], while the relief of not touching a spider actually conveys a safety cue. We hypothesize that the actual approach behaviour of the feared cue without experiencing a catastrophe is responsible for the process of memory reconsolidation, but this conjecture has not yet been critically scrutinized. Another drawback of the current intervention involves the feasibility of the false information regarding the instruction to touch a spider, given that this information is easily accessible via the Internet.

A further consideration is that it remains unknown whether a similar procedure would also be effective for patients who are suffering from severe levels of spider fear, as the intervention was originally developed for a subclinical fear of spiders. Fear-conditioning studies showed that stronger fear memories require longer or multiple reactivations in order to induce memory reconsolidation [52]. Likewise, in a new series of pilot cases we discovered that some of the patients with a clinically diagnosed spider phobia (DSM-5, APA) [60] required a different reactivation procedure from that in our previous study. As long as no independent index is available to assess memory reconsolidation in clinical practice, systematically testing different reactivation procedures might contribute to uncovering the optimal and boundary conditions for inducing memory reconsolidation in clinical practice. Currently, we are testing different memory reactivation procedures in patients with spider phobia and other specific phobias; some cases are extremely successful, while others actually fail and do not convincingly profit from the reconsolidation intervention [52].

Difficulties such as these are even more crucial when targeting more complex emotional memory disorders in patients with PTSD, given that the timing of reactivating trauma memory is under less experimental control than in specific fears or phobias [23]. Although preliminary evidence in patients with PTSD revealed a reduction in a trauma-relevant physiological response following a reconsolidation intervention [19–21], these studies did not include the crucial control conditions for demonstrating memory reconsolidation (i.e. memory reactivation with placebo, and propranolol without memory reactivation). Moreover, the initial positive effect of a reconsolidation intervention for trauma memory could not be replicated in three follow-up trials testing different amnestic agents [22]. It bears mentioning, however, that the design of the previous treatment studies raises several questions with respect to the necessary conditions for a reconsolidation intervention. The success of a reconsolidation intervention clearly depends on two conditions: (i) memory reactivation should trigger *destabilization* of the fear memory, and (ii) the amnestic drug should disrupt the *restabilization* of the fear memory. In the previous clinical trials, script-driven imagery was used for the reactivation of the trauma memory, even though this method has been explicitly developed to measure retrieval of the trauma memory and *not* reconsolidation. In light of the observations in fear-conditioning research that retrieval is not sufficient to trigger memory reconsolidation [6–8], such a reminder session is probably ineffective in inducing the process of memory reconsolidation. Furthermore, the systemic administration of a low dose (10 mg) of short-acting propranolol HCl 90 min before memory reactivation, combined with a low dose (20 mg) of long-acting propranolol HCl immediately prior to script-driven imagery is not theoretically grounded. If upon memory reactivation, propranolol targets the process of memory reconsolidation, the β-ARs are presumably involved during a specific time window after memory reactivation [39]. We have recently tested the timing of drug administration and discovered that propranolol was effective when systemically administered 90 min or 1 h before memory reactivation, immediately after [4–13], and 1 h after memory reactivation. When given 2 h after memory reactivation, propranolol was no longer effective [5]. Hence, by exploiting the pharmacokinetic signature of propranolol (*T*_peak_ = 1–2 h; *T*_1/2_ = 5 h), we identified a delayed and specific time window (less than 4 h post-reactivation) during which β-adrenergic receptors are decisively involved in the reconsolidation of fear memory. This observation seems to be in line with observations in animal studies where peaks in noradrenalin 1–2 h after (re)learning are related to the reconsolidation of memory [39]. For a proper translation to clinical practice, insights on the necessary and boundary conditions to induce memory reconsolidation in the laboratory should be taken into account.

In summary, before strong conclusions can be drawn as to the relevant features of a reactivation procedure to modify maladaptive memories, a systematic search into reconsolidation interventions is warranted. In concert with efforts to demonstrate fear-reducing effects of pharmacological memory manipulations, alternative approaches to target memory reconsolidation have been developed as well. From these non-pharmacological approaches, the reactivation–extinction procedure has been most extensively studied [61]. Drawing upon the idea that reconsolidation serves as a means of updating memory under changing environmental conditions [55], it has been suggested that extinction training after a brief reminder might serve to incorporate the inhibitory learning into the reactivated memory trace [62]. Unlike the pharmacological amnestic interventions, the reactivation–extinction procedure has not been widely replicated thus far [61,63]. Another consideration regarding the reactivation-extinction procedure is that superior extinction learning rather than the process of memory reconsolidation could also explain the fear-tempering effects [63]. Irrespective of the manipulation, the delicate transitions between the different memory processes (i.e. retrieval, reconsolidation, limbo and extinction) pose a huge challenge to clinical research on memory reconsolidation. In pursuing ways to overcome the putative boundary conditions on triggering memory reconsolidation, collaborative efforts between animal and human researchers are essential. In addition to a trial and error approach, future research should aim to uncover a real-time index for memory reconsolidation, which would enable an independent test of the boundary and optimal conditions of a memory reconsolidation intervention in clinical practice.

## 6. Conclusion

Basic science in animals and humans suggests that we are on the verge of a breakthrough in reconsolidation-based interventions, but the critical conditions for targeting the complex and pervasive fear memories typically encountered in clinical practice are yet unknown. Translating basic findings from animal literature to clinical trials without a full understanding of the mechanisms of change very often leads to disappointing and confusing results. Alternatively, as most often seen in clinical psychology and psychiatry, treatments emerge from the field of therapy, with researchers subsequently dissecting what may be effective about the interventions. Dozens of treatment effects studies have appeared in the literature showing that different intervention techniques are either more or less effective. Research at the applied level has important social and policy significance, but it is ill-suited for elucidating the determinants and mechanisms of change. Large-scale clinical trials provide the type of data that patients and decision-makers need to choose among alternative modes of treatment.

In the quest for a means of therapeutic forgetting, a transdiagnostic perspective with a focus on the neurobiological principles of emotional learning and memory is proposed, rather than orienting toward the DSM with the focus on isolated diagnostic categories of mental disorders. While basic science and clinical science are generally separate worlds, integrating these disciplines in a bi-directional translational heuristic may foster our understanding of the mechanisms of change in the treatment of emotional memory disorders. Insights from basic science may shape clinical interventions, and vice versa, clinical observations, preferably from small-scale case series, may fuel basic science, with the eventual aim to decipher the dynamic interplay between the plasticity and stability of emotional memory.

## Endnote

1 Actually this was a rediscovery. Post-reactivation transient amnesia was first revealed in the 1960s by Donald Lewis.

## Data accessibility

This article has no additional data.
